# The micropolitics of implementation; a qualitative study exploring the impact of power, authority, and influence when implementing change in healthcare teams

**DOI:** 10.1186/s12913-020-05905-z

**Published:** 2020-11-23

**Authors:** Lisa Rogers, Aoife De Brún, Sarah A. Birken, Carmel Davies, Eilish McAuliffe

**Affiliations:** 1 University College Dublin Centre for Interdisciplinary Research, Education, and Innovation in Health Systems (UCD IRIS), University College Dublin School of Nursing, Midwifery and Health Systems, Dublin, Ireland; 2 Department of Implementation Science, Wake Forest School of Medicine, Winston-Salem, Noth Carolina USA

**Keywords:** Micropolitics, Power, Authority, Influence implementation science, Healthcare, Teams, Context

## Abstract

**Background:**

Healthcare organisations are complex social entities, comprising of multiple stakeholders with differing priorities, roles, and expectations about how care should be delivered. To reach agreement among these diverse interest groups and achieve safe, cost-effective patient care, healthcare staff must navigate the micropolitical context of the health service. Micropolitics in this study refers to the use of power, authority, and influence to affect team goals, vision, and decision-making processes. Although these concepts are influential when cultivating change, there is a dearth of literature examining the mechanisms through which micropolitics influences implementation processes among teams. This paper addresses this gap by exploring the role of power, authority, and influence when implementing a collective leadership intervention in two multidisciplinary healthcare teams.

**Methods:**

The multiple case study design adopted employed a triangulation of qualitative research methods. Over thirty hours of observations (Case A = 16, Case B = 15) and twenty-five interviews (Case A = 13, Case B = 12) were completed. An in-depth thematic analysis of the data using an inductive coding approach was completed to understand the mechanisms through which contextual factors influenced implementation success. A context coding framework was also employed throughout implementation to succinctly collate the data into a visual display and to provide a high-level overview of implementation effect (i.e. the positive, neutral, or negative impact of contextual determinants on implementation).

**Results:**

The findings emphasised that implementing change in healthcare teams is an inherently political process influenced by prevailing power structures. Two key themes were generated which revealed the dynamic role of these concepts throughout implementation: 1) Exerting hierarchical influence for implementation; and 2) Traditional power structures constraining implementation. Gaining support across multiple levels of leadership was influential to implementation success as the influence exercised by these individuals persuaded follower engagement. However, the historical dynamics of each team determined how this influence was exerted and perceived, which negatively impacted some participants’ experiences of the implementation process.

**Conclusion:**

To date, micropolitics has received scant attention in implementation science literature. This study introduces the micropolitical concepts of power, authority and influence as essential contextual determinants and outlines the mechanisms through which these concepts influence implementation processes.

**Supplementary Information:**

The online version contains supplementary material available at 10.1186/s12913-020-05905-z.

## Background

Healthcare systems are complex, inherently political structures. Although often viewed as one large cluster, healthcare organisations are social enterprises encompassing shifting coalitions of interest groups [1]. Healthcare delivery has evolved from care by one all-knowing physician to the provision of care by multidisciplinary teams (MDTs) [2]. MDTs are characterised by numerous healthcare professionals (HCPs), from several disciplines interacting in highly unpredictable environments to optimise patient care [3–5]. By valuing the skills and knowledge of each discipline, holistic patient-centred care can be achieved [6, 7]. However, interprofessional collaboration is challenging. Each professional group has a unique identity that corresponds to their discipline-specific training and clinical experience [8, 9]. This identity means that despite sharing the same goal of improving patient outcomes, HCPs have differing priorities, roles, and expectations about how care should be delivered [10–12]. These divergent interests often cause HCPs to work within discipline-specific silos (nursing, medicine, allied health) [10]. Furthermore, hospital managers represent another stakeholder group with additional priorities. To achieve financial and efficiency targets these individuals guide strategic planning and regulate the resources available to HCPs [13].

To reach agreement among these multiple stakeholders and achieve safe, cost-effective patient care, negotiating the micropolitical context of healthcare is a common experience for MDTs [14, 15]. Politics has been defined as negative self-serving behaviours or natural organisational processes [14]. Within this study, politics refers to the use of power, authority, and influence to affect team goals, vision, and decision-making processes [16]. Pfeffer [17] argues that power is obtained through the skilful use of political tactics. Power is also regarded as the ability to exercise political influence to achieve desired outcomes [18, 19] . Thus, power and politics are intertwined concepts which play important roles during interpersonal interactions within organisations [20]. While power has been conceptualised as a possession (i.e. exercising power over others) [21], this study assumes a post-modernist perspective, describing power as a relational force existing between two or more people [22]. By adopting this perspective, power can be enacted by all. This perception is evident in implementation science literature, with peers cited as an accessible and convincing influence to persuade staff enthusiasm for change [23]. However, traditional norms of organisations reinforce staff identities meaning the voices of some team members are valued more than others [24].

Within healthcare, a hierarchical power structure has historically been adopted with physicians typically assuming dominant roles [12, 25, 26], while other professions encounter challenges establishing their status in patient care decisions [27, 28]. It is suggested that the position of a discipline within a team hierarchy influences how emotions such as fear are experienced [29]. Although the relationship between nurses and doctors has evolved, nurses continue to struggle for autonomy with physicians remaining the primary decision-makers in practice [30, 31]. Additionally, while the emergence of allied healthcare professionals (AHPs; physiotherapists, occupational therapists, social workers, dietitians, speech and language therapists, pharmacists) has improved the range of services available to patients, these professions too have traditionally assumed subordinate positions within MDTs, often observed as “allied to medicine” [32]. However, despite dominating clinical decision making, the introduction of managerialism in healthcare has challenged physician autonomy. Management authority in hospital decision-making has reduced physician capacity to ensure strategic decisions benefit their professional interests [13].

The micropolitics surrounding healthcare teams are influential mechanisms for cultivating change [33]. The diverse values held by each profession implies that the consequences of implementation may not be uniformly positive for all disciplines. This threat to existing norms often triggers resistance among staff [15, 19, 34]. However, political skill enables change agents to account for diverse professional interests and effectively exercise their influence to mediate collective action [20, 35, 36]. Therefore, politics is a mechanism for creating order [37]. Employing political influence gives meaning to a change effort [37] and enhances staff trust in the new reform [35]. Consequently, political skill reduces the uncertainty associated with change, enhancing the likelihood for successful implementation [35]. Despite the importance of the micropolitical context, the concepts of power, authority, and influence have received scant theoretical or empirical attention in implementation science [35, 38]. Additionally, although teams are central to the organisational structure of healthcare, there is a poor understanding of team level contextual determinants within the field [39]. This study addresses these gaps by exploring the mechanisms through which micropolitics influences the implementation of a team-based leadership intervention.

## Methods

### Study background

This study focuses on one facet of the analysis from a wider body of research which examined the active role of context during the implementation of a collective leadership (CL) intervention. The objective of this intervention was to introduce CL to MDTs using a suite of educational sessions to improve team performance and safety culture [40] (Supplementary file 1). The CL intervention was piloted with four heterogeneous healthcare teams over a one-year period. Supplementary file 1 also provides a reflexive account, detailing researcher characteristics and potential biases.

### Study design

The multiple case study design adopted enabled an in-depth investigation of context without involving explicit control of the healthcare settings of interest i.e. naturally occurring MDTs. This design allowed researchers to preserve the meaningful characteristics of the team and their interactions [41]. For this research, a ‘case’ was defined as the implementation of a CL intervention in one MDT. Aligned with Yin’s [41] interpretation of a “good case study”, this research used a triangulation of qualitative research methods. Observation and interview data revealed “what goes on” in each team, while also eliciting insider descriptions of the context [42].

### Study sample

Two of the four teams introducing the CL intervention were chosen as implementation case studies due to their divergent case characteristics (Table 1). Since observations were completed at monthly preparation meetings and intervention sessions, sampling relied on staff availability which varied depending on workload, staffing levels, and shift patterns (e.g. night duty). For interviews, a purposeful sample of participants were recruited from a diverse range of disciplines with varying levels of engagement throughout implementation (Table 2). Due to the continuous rotation of staff, junior doctors, and multi-task attendants (duties include cleaning, and catering services) were absent from the sample. However, sample adequacy was achieved as during the interview process, a sufficient depth of information was gathered to produce no new information.

**Table 1:** Case characteristics

	Case A- Willow	Case B- Brickley
**Hospital classification**	Model 3- Hospitals that can provide 24-h acute surgery, acute medicine, and critical care.	Model 4- provide tertiary and supra-regional care in addition to 24-h acute surgery, acute medicine, and critical care.
**Location**	Rural	Urban
**Financial and Governance Structure**	Statutory hospital- funded and governed by the national government agency, the Health Service Executive.	Voluntary hospital- acquires greater autonomy as owned by a religious order and subsequently reports to a hospital board rather than the Health Service Executive. This hospital type also receives funding from the state.
**Hospital size**	Approximately 200 bed capacity	Approximately 600 inpatient bed capacity, 85-day bed capacity
**Team size**	*n* = 65	*n* = 73 Team divided across two wards which are located on different levels of the hospital. The nursing staff work permanently on one of the wards while the medical team and the allied healthcare professionals (AHPs) move between units.
**Team speciality**	Surgical	Medical
**Team stability**	• Intern: 3-month rotation • Senior House Officer: biannual rotation • Registrar: biannual/annual rotation • AHPs: biannual rotation • Multi-task attendants: 3-month rotation	• Intern: 3-month rotation • Senior House Officer: biannual rotation • Registrar: biannual/annual rotation • Junior AHPs:4–6-month rotation
**Team culture (prior to implementation)**	• Hierarchical- within this team some participants felt intimidated or overlooked by their senior colleagues.	• Collective- the team characterised its culture as open, inclusive, and multidisciplinary. However, a divide was acknowledged between the two wards which was recognised as impacting the relationships among staff.

**Table 2:** Characteristics of interview participants

Case	Participant	Sex	Sessions attended	Sample details
**Case A (Willow)**	Nurse1W	F	3	Sample included registered nurses, and clinical nurse managers
	Nurse2W	F	4	
	Nurse3W	F	2	
	Nurse4W	F	0	
	Management1W	F	8	Sample incorporated senior managers of the organisation
	Management2W	F	8	
	Medic1W	M	5	Sample comprised of senior physicians (consultants and registrars)
	Medic2W	M	5	
	Support Staff1W	M	2	Sample encompassed the views of healthcare assistants (staff who assist with bedside care e.g. bathing, feeding patients)
	AHP1W	F	2	Sample contained various disciplines from the field of allied health
	AHP2W	M	6	
	AHP3W	F	3	
	AHP4W	F	4	
**Case B (Brickley)**	Nurse1B	F	3	Sample included registered nurses, advanced nurse practitioners, and clinical nurse managers
	Nurse2B	F	1	
	Nurse3B	F	2	
	Nurse4B	F	7	
	Nurse5B	M	6	
	Nurse6B	F	4	
	Medic1B	F	7	Sample comprised of senior physicians
	Medic2B	F	4	
	Support Staff1B	M	1	Sample encompassed the views of healthcare assistants
	AHP1B	F	6	Sample contained various disciplines from the field of allied health
	AHP2B	F	4	
	AHP3B	F	1	

### Data collection

To understand the effect of contextual factors on implementation success, the Consolidated Framework for Implementation Research (CFIR) [23] and Proctor et al.’s implementation outcomes [43] guided the development of the observation template (Supplementary file 2) and interview guide (Supplementary file 3). When translating evidence into real-life contexts, it is crucial to understand whether an intervention’s failure is due to an ineffective intervention or whether a potentially effective intervention was deployed incorrectly [43]. Proctor et al. [43] provides a taxonomy that clearly differentiates implementation outcomes from service and patient outcomes. Theories, models, and frameworks also provide greater insight into the mechanisms of implementation [44, 45]. Consequently CFIR, a widely operationalised meta-theoretical framework that aids in the classification of contextual determinants [46–49] was used to inform data collection.

#### Observations

Observation is invaluable for providing insights into everyday practice that would not be achieved through other data collection methods [42]. Throughout this study, the researcher assumed a “peripheral membership role” [50], establishing a rapport with each team but staying sufficiently detached to maintain an “outsider” perspective [51]. Thirty-one hours of observations were completed between January and November 2018. Handwritten field notes were taken during each observation, which included phrases and quotations relating to participants’ dialogue, interactions, and physical surroundings. These notes were transcribed into detailed accounts within 24-h of each site visit to ensure the thorough recounting of observed events. To evaluate the intervention’s implementation, these field notes were then inputted into an observation template which was developed for the purpose of this research (Supplementary material 2).

#### Interviews

Following the intervention’s implementation, semi-structured interviews were conducted at both sites in February and March 2019. By eliciting a greater understanding of participant experience, these data assisted in identifying contextual factors influencing successful implementation from the perspectives of those involved. The interview schedule was piloted once, resulting in minimal changes to the structure. This pilot interview was included in the final dataset. Twenty-five participants were interviewed once, and interviews ranged in duration from 18 to 57 min (average 38 min). All interviews were audio-recorded and transcribed verbatim (see Supplementary material 3 for topic guide).

### Data analysis

An iterative approach to data analysis was adopted in which data collection and analysis were concurrent rather than successive [52, 53]. Throughout implementation a context coding framework succinctly collated the data sources into a visual display (Supplementary file 4) [54]. Although this approach offered a high level overview of implementation effect (i.e. the positive, neutral, or negative impact of contextual determinants on implementation), further analysis was required to understand the mechanisms through which context influenced implementation success [54]. Thematic analysis as outlined by Braun and Clarke [55], guided the analysis structure. Rather than applying a prescriptive list of CFIR domains or implementation outcomes, an inductive approach to coding was chosen to ensure themes strongly reflected the data collected. To generate a more complex understanding of the results, the data were double coded [56]. LR analysed the complete dataset, while ADB double-coded a random 10% of data. The process aimed to challenge researcher assumptions and facilitate a more complex, in-depth understanding of the data collected [56]. In addition, to highlighting new insights, the process enhanced the trustworthiness of the findings as a high level of agreement was informally demonstrated when comparing and discussing the researchers’ coding patterns. NVivo 11 supported the analysis process [57].

### Ethics

Favourable ethical opinion was obtained from the University College Dublin Research Ethics Committee (ref: HREC-LS-16-116397) and the participating hospital sites. All participants provided written informed consent during each phase of data collection and all potentially identifiable characteristics were removed from each transcript to maintain anonymity.

## Results

Both teams successfully completed the CL intervention, implementing the required eight intervention sessions and achieving consistent attendance throughout implementation (average attendance for both cases = 12 participants). The utility of the intervention also inspired both teams to engage in service improvement initiatives to respond to problems raised during the team sessions. However, the reach of the intervention was only partial with some team members failing to engage with the intervention’s implementation.

The inductive analysis revealed that implementing change in healthcare teams is an inherently political process influenced by prevailing power structures. A traditional hierarchical system exemplified by leaders’ downward influence on followers through formal authority was evident in both cases. However, the influence of this hierarchy on implementation differed across settings. Two key themes were generated from the data; 1) Exerting hierarchical influence for implementation; and 2) Traditional power structures constraining implementation. To maintain participant anonymity, pseudonyms have been assigned to both cases. Table 1 presents these pseudonyms with additional information to assist the reader in contextualising the findings.

### Exerting hierarchical influence for implementation

Senior leaders and managers from each case played a fundamental role in implementing the CL intervention. By exerting their authority, these staff stimulated engagement and endorsed the relevance of the intervention among team members. Sub-themes in this section are organised to explain the influence of senior physicians, senior managers, and middle managers on implementation.

#### Physician rule

Willow was indicative of an explicit hierarchy, characterised by silo working, authoritarian leadership, and fear. Within this setting staff associated fear with their inability to speak up which was often related to their interpersonal interactions with some senior physicians within the team. Senior physicians were acknowledged as the most powerful influence on the team, described as “next to God” (AHP2W), “a step above” (Management2W), and “untouchable” (AHP3W). One participant implied that physician power was due to organisation’s rural location and the associated challenges of retaining staff. Others related this influence to the central role senior physicians possess in patient care delivery, characterising them as “ultimately responsible” (Nurse2W). Comparable to Willow, the senior physicians of Brickley appeared to establish team culture. Brickley was noticeable for its “inclusive” (AHP3B) approach. Some participants suggested that this cultural difference was related to senior physician specialities. For staff within both sites, a hierarchical approach to decision making was associated with surgical teams.

“The focus is so heavily on surgery and the surgical doctors…a kind of God complex that is difficult to breakdown” (AHP3B)

The power dynamics established in each case had a significant impact on the intervention’s implementation. Reflecting their authority, senior physician support was acknowledged as crucial for ensuring the intervention’s adoption.

“If you can convince the consultants {senior physicians} …then you will get everybody on board” (Medic2W).

Within Brickley, senior physician support for the intervention was strong and highly visible. One senior physician was “heavily involved” (Nurse6B) with implementation; organising and delivering intervention sessions and “exhort{ing}” the value of attendance (Medic1B). Given the influence of this senior physician, their commitment was acknowledged as important for enhancing engagement by establishing the intervention’s relevance. Although another senior physician simply attended and contributed to intervention sessions, this support was also recognised as influential to reinforce the intervention’s legitimacy.

“They were there you know… role modelling …it’s not do as I say, it’s do as I do” (Nurse5B).

In contrast, Willow’s senior physicians failed to engage throughout implementation, with only one of the four senior physicians attending intervention sessions. Participants suggested workload, competing priorities, and a perceived intolerance for the “soft stuff” (i.e. the CL intervention) as potential explanations for their inadequate engagement (Observation6W).

#### Senior manager authority

Willow’s senior management were “strong supporters” (Management2W) of the intervention. Without this top-down support organising and facilitating the intervention, participants believed the intervention would have failed. However, compared to senior physicians, Willow’s senior managers were perceived to have less influence in ensuring staff engagement. Attendance at the intervention appeared to vary in accordance with senior physician support (i.e. when senior physicians were present, greater staff engagement was achieved). Conversely, senior management needed to “chase people” throughout implementation to guarantee engagement (Observation15W). As implementation progressed, the difficulties in engaging staff became frustrating for senior management, which impacted the feasibility of sustaining the intervention; you get “spun out trying to get people to come, it’s like pulling teeth” (Observation 16 W).

For senior staff in Brickley, organisational support promoted the importance of the intervention, and empowered staff to recognise that “{they} were a team worth supporting” {Medic1B). Like Willow, frontline staff (i.e. doctors, nurses, AHPs working on the wards) within Brickley were “volunteered” by senior management to participate in the intervention’s implementation (Nurse6B). However, in Brickley, staff appeared to have less familiarity with their hospital management. This distant relationship was evidenced by frontline staff perceptions of senior management support during implementation. Most participants equated senior managerial support simply to the provision of refreshments during the intervention, while others were unaware of any senior management engagement. Most team members felt the remote support was appropriate due to the “ward-based” nature of the intervention (Medic2B). However, for one participant, this distant engagement was disappointing as the perceived value of the intervention was diminished because the team were left “fend for {themselves”} (AHP1B).

#### Middle manager influence

Clinical nurse managers (CNMs) also played a significant role in implementing the CL intervention at both sites. CNMs alluded to using their typical role as the “middle-man” (Observation11W) to transfer information about the project across professional groups. Other important responsibilities of CNMs included directing people to attend the intervention and leading sessions. However, throughout implementation, the CNM of one ward in Brickley did not engage with the intervention. This lack of support led to the poor dissemination of information about the intervention and its outputs among nursing staff. This impacted staffs’ understanding of the initiative, which subsequently influenced the intervention’s acceptability and adoption among these nurses.

### Traditional power structures constraining implementation

In addition to influencing staff engagement and the perceived credibility of the intervention, the power of senior leaders also generated a culture of fear, silence, and isolation within teams. The subthemes of 1) *Perpetuating a culture of fear*; 2) *A chain of forgotten voices*; and 3) *The silo effect* demonstrate how perceptions of influence can impact staff experiences of implementation.

#### Perpetuating a culture of fear

Throughout implementation, Willow’s staff described a “put up or shut up” culture within the team (Observation8W). This culture was illustrated by the evocative language used by team members. Participants revealed how “you learn the hard way not to open your mouth” (Observation10W). Staff explained how this hierarchy results in them feeling “guarded” (AHP2W), “constrained” (Observation5W), and “beaten down” (Nurse2W). Yet, this behaviour appeared to be overlooked due to the “skills” senior physicians offer the hospital (Observation10W) and the perceived threat of them leaving the organisation, given the challenges in recruiting, and retaining staff. Some participants held the defeatist attitude that this hierarchical mind-set would only change when these senior physicians retired, and a new mind-set replaced them. An observed difference in how staff behaved towards senior physicians as compared to other team members was noted in the use of professional titles rather than first names. To reduce the disparity between professions, ground rules were established to remove the use of titles in preference of first names when communicating during the intervention (Observation6W).

Comparable to Willow, some staff in Brickley perceived a “rank” of influence within the team (Support staff1B) which reduced staff’s perceived psychological safety. Within this context, the ability of team members to speak-up and express their beliefs was influenced by senior management presence at the intervention. Although attendance by senior managers was customary at Willow, for Brickley, their presence at two sessions inhibited the engagement of some staff. Team members suggested that senior management attendance silenced the team. The relationships between frontline staff and senior management may reflect the diverse organisational cultures of each case. However, these relationships may also be attributed to the differences in organisational size. Frontline staff within the smaller, regional hospital depicted a close relationship with senior management which explained the acceptability of management presence throughout implementation. However, senior management in the larger, urban organisation were not considered part of the team, therefore, their attendance was considered inappropriate.

“You don’t see {senior management} very often…you’re not actually working with them day in, day out… It was nice just having your own team” (Nurse4B).

#### A chain of forgotten voices

The chain of command evidenced in both cases appears to render some staff without a professional voice. While senior physicians resided “at the top” (AHP4W) of Willow’s hierarchy, other HCPs considered themselves “removed” from the team (Observation5W). Willow’s AHPs appeared unable to exert their professional opinion in some circumstances; “I know my place…who speaks and who listens…” (AHP3W). These power differences between professions impacted the perceived appropriateness of the intervention among staff. Due to their position in the hierarchy, Willow’s AHPs emphasised the relevance of the intervention given its aim to promote a more inclusive culture. AHPs recognised the initiative as an opportunity to improve their position and become core members of the MDT. Thus, their satisfaction with the intervention was enhanced, leading to their consistent attendance throughout implementation. Nurses from both cases, too, felt their influence on decision making was limited and considered the intervention valuable in enabling “them to have a voice” (Management2W) and “feel sometimes you’re listened to” (Nurse3B).

However, the adoption of the intervention among the support staff (e.g. healthcare assistants who assist with bedside patient care) of each case was poor. These staff members perceived the intervention as irrelevant to their role within the team. Support staff believed that a hierarchical “ladder” existed (Support Staff1B), suggesting that the intervention was more advantageous for those “higher up” (Support Staff1W), listing doctors, nurses, and management as possible beneficiaries. The adoption of the intervention by support staff was poor due to the perceived irrelevance of the intervention content.

#### The silo effect

Silo working was evident within both cases, with disciplines working in isolation rather than collaboratively. Willow’s staff openly acknowledged how “everybody does their own thing” (AHP1W) within and across disciplines. Aligned with the team’s established hierarchy, “a big divide” (Nurse1W) was perceived between senior physicians and other professions, leading to communication “fences” between disciplines (Medic1W). Although not explicitly mentioned by most, some staff of Brickley alluded to silo working when outlining the benefits of the intervention; “took people out of their silos and mixed us all around” (Medic1B). However, two team members from different professions reported a clique within Brickley. This perhaps reflects staff members’ position within the team hierarchy, where silo working is more noticeable depending on their role. In addition to interdisciplinary segregation, a division across the wards of Brickley was apparent; “there’s definitely a ‘we work down here, they work up there {attitude}’” (Nurse1B).

The effect of this silo working on implementation was evidenced in the dissemination of the intervention and its subsequent acceptability and adoption. Successful dissemination appeared reliant on the support of senior team members within each professional group. A senior physician explained that they “don’t have the remit to tell nursing, AHPs, or whatever else” (Medic1B), but instead can disseminate information within the medical team. Additionally, due to the division of Brickley into two wards and the poor engagement of one CNM, staff from one ward were dependent on a roving manager (i.e. spread across several locations) to disseminate information as they were passing through the unit. This inadequate exposure to the intervention led to a lack of nursing staff engagement with the initiative from this ward. Comparable to Brickley, although some staff within Willow reported enhanced team functioning following the intervention’s implementation (e.g. 50% reduction in surgical discharges after 5 pm), others who did not consistently engage remained unaware of the team’s achievements. This localised understanding of accomplishments likely impacted the acceptability and perceived appropriateness of the intervention for all team members subsequently influencing the intervention’s wide-spread adoption across the team.

## Discussion

**Fig. 1:**
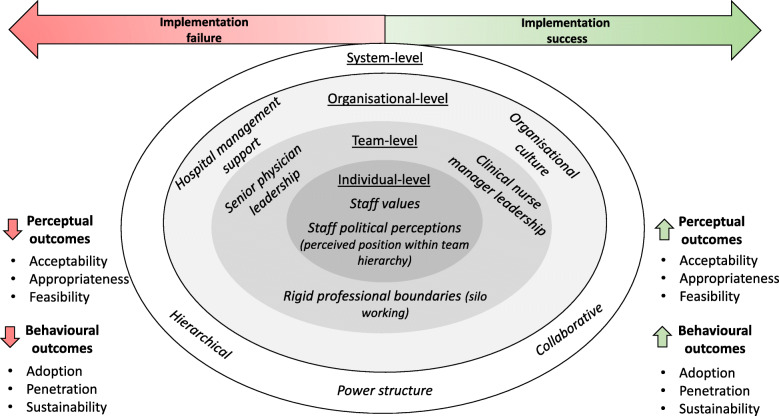
Conceptual Framework of the impact of politics and power on implementation success

Using a multiple case study design and a triangulation of qualitative research methods, this study explored the role of power, authority, and influence when implementing a CL intervention among two MDTs. The findings demonstrate that implementing change in healthcare is an inherently political process, heavily influenced by established power structures. This paper demonstrates the need to account for the micropolitical context when implementing change. Gaining support across multiple levels of leadership was influential to implementation success as the influence exercised by these individuals persuaded engagement. However, the historical context of each team (and organisation) determined how power was perceived and negotiated. This in turn negatively shaped experiences of the implementation, impacting implementation outcomes. By collating the extant literature (e.g. [3, 9, 15, 58, 59]) with findings from this research, a conceptual framework has been developed to capture how politics and power can impact implementation success (Fig. 1). This framework acknowledges that team micropolitics are interdependent with other levels of the health system (i.e. individual, organisational, system). Figure 1 defines implementation success through staffs’ perceptual (acceptability, appropriateness, feasibility) and behavioural (adoption, penetration, sustainability) responses to a change effort. This framework emphasises that the interplay between political constructs will either drive or impede an implementation effort. For example, although the influence of hospital management would likely support the adoption of change, if rigid professional boundaries exist within a team and if an intervention contradicts staff values, an implementation effort will likely fail. By summarising the multidimensional impact of power, authority, and influence across system levels, this framework can be used to support the development of implementation strategies when introducing change in healthcare practice. If change agents recognise rigid professional boundaries within teams, perhaps a cross-disciplinary approach to implementation would be beneficial to enable MDT members to discuss, dispute and establish the utility of an intervention [60, 61].

From a critical perspective, leadership is described as the art of persuading people to work towards a common goal [62]. The extant literature also depicts politics as a means of resolving conflict through a process of bargaining, negotiation, and compromise [14, 36, 37]. These actions are often described as management activities [14, 36, 63], meaning political behaviour is an unseen, unacknowledged characteristic of effective leadership. Consistent with the extant literature [58, 59, 64], this study confirms that leaders at multiple levels of an organisation can enhance implementation (Fig. 1). The findings also provide a novel insight into the effect of this influence when introducing change within healthcare teams.

The findings support the extant literature which identifies senior physician engagement as a critical feature of implementation success [65–68]. Implementation success is considered to be dependent on the compatibility of the initiative with physician values and whether these physicians perceive a need for change [67]. This study confirms that senior physician attitudes toward an impending implementation will determine whether the team accept and adopt an innovation [69]. Within Willow’s hierarchical context, the CL intervention which challenges traditional power structures was ignored by most physicians. However, due to their more collaborative approach, the physicians of Brickley were highly supportive of this inclusive innovation. The hierarchical authority of these senior physicians [10, 30, 32] enabled them to promote their values and negotiate a response from each team that aligned with their interests. Therefore, senior physicians function as gatekeepers to innovation, influencing the engagement and wide-spread adoption of interventions across MDTs.

This study also confirms the important role of management throughout implementation [1, 66, 68, 70, 71]. Management promote the values of the organisation which means their support is influential to normalise a new practice among staff [24]. Both cases received organisational support, however, the strength of this support and its influence on implementation varied across settings. Within Brickley, although senior management involvement with implementation was minimal, their distant support enhanced the commitment of senior MDT members. Within Willow, senior management engagement with implementation was strong. Through leading by example, the credibility of the intervention was heightened for most frontline staff [59] which enhanced engagement with the intervention [68, 70]. However, despite active managerial support, Willow’s senior physicians remained reluctant to participate in the intervention’s implementation. This may reflect senior physician resistance to engage in management-initiated improvement initiatives due to a perceived threat to autonomy [13, 59, 72, 73]. Identifying these power dynamics is pivotal in determining the level of involvement required by each stakeholder (represented in Fig. 1) throughout implementation.

Obtaining the support of middle management (e.g. CNMs) was also considered fundamental, with staff primarily looking to their supervisors for guidance on how to respond to change [69, 74, 75]. The network centrality of these managers connects the operational core of an organisation with senior management [63]. Due to this unique position, middle managers mediate the conflicting needs, demands, and priorities of stakeholders above (senior management) and below (frontline staff) their position in the team hierarchy [76, 77]. However, this role also functions as a mechanism for change. By understanding the strategic and clinical priorities of multiple stakeholders, middle managers can gather, synthesise, and adapt information received from senior management and disseminate it appropriately to assure its utility across interest groups. Therefore, middle managers shape the team’s collective understanding of the intervention, which can stimulate or discourage acceptance for change [63, 76]. While the role of the CNM during implementation has previously been described as passive [78], this research exemplifies that CNMs’ support for implementation is vital to promote information about the intervention. CNMs are consistent points of communication and dissemination for all professions [79, 80]. This research demonstrates the boundary-spanning role of CNMs and highlights their influence on collective sensemaking within MDTs.

Although the influence of senior leaders positively affected implementation, the prevailing power structures also resulted in varying MDT responses to the intervention. The power disparities observed in both cases enhanced the commitment of some staff to implementation as the intervention was perceived as an opportunity to improve their position within the team. However, enthusiasm for the intervention was not universally observed among participants. Support staff (e.g. healthcare assistants) from each site perceived the intervention as irrelevant to their professional role. This impression likely reflects their position within the team hierarchy. Healthcare assistants have reported feeling undervalued in their role, viewed as the team “workhorses” [81]. Therefore, factors such as an individual’s job responsibilities and perception of their place in the hierarchy will influence their interpretation of workplace politics [20, 33, 82]. Job satisfaction, organisational commitment, and job performance have been listed as consequences of political perceptions [33]. However, this study provides a novel insight into the impact of political perceptions on implementation success. As outlined in Fig. 1 stakeholders’ perceptual (e.g. acceptability) and behavioural (e.g. adoption) responses to implementation depend on where staff position themselves within the team hierarchy (i.e. the intervention’s perceived acceptability is contingent on staff’s role within the team which promotes/limits the intervention’s perceived utility and subsequent adoption).

The findings also reveal how silo working in MDTs can impact staff experiences of implementation. Foucault [22] argues that knowledge and power are intertwined concepts. Within healthcare, staff use discipline-specific knowledge to create boundaries around their professional identity, strengthening their voice within the MDT [12]. However, the historical power dynamics between professions determines whether professional opinions are valued by other MDT members [12]. Previous literature has identified professional tribalism as a barrier to effective communication, inhibiting the delivery of optimum patient care [10–12]. This study demonstrates that staff reliance on intraprofessional communication also impedes implementation by limiting staff understanding of the initiative, impacting adoption. Social identity theory may explain this failure to interprofessionally share information. Social identity theory suggests that individuals form groups based on compatible social factors such as professional affiliation (e.g. nursing) [8]. Members of the same group are inclined to promote the opinions of fellow group members while devaluing the views of those outside the group. Comparable to Ferlie et al.’s [9] findings, this research further reveals how ingroup cohesion and outgroup discrimination can impact the adoption and spread of an intervention across professional boundaries. Despite the intervention receiving consistent support from physicians and AHPs, the poor commitment of a CNM in one ward in Brickley, limited nurse engagement with the intervention from this unit. Therefore, tribalism stimulated by cultural, political, and institutional socialisation impacts how an intervention is promoted and perceived across MDTs (see Fig. 1).

While this article offers new insights into the mechanisms through which the micropolitical context of MDTs impacts implementation, some limitations should be noted. The generalisability of the findings is limited due to the use of two cases. However, the thick descriptions presented in this study enhances the ecological validity of the results as readers can determine whether the findings are applicable to their setting [83, 84]. The Hawthorne effect may have impacted data collection. However, as only one researcher observed all intervention sessions, this limitation was likely diminished as the effect of an observer is recognised to lessen over time [85, 86]. Additionally, although a diverse sample of HCPs were recruited, the perceptions of some MDT members were absent due to the continual rotation of staff. However, by using multiple sources of data, some of these views are accounted for within the final data set. Finally, to mitigate potential researcher bias, a reflexive journal was maintained, and all researchers were involved in the analysis throughout the evaluation process.

Despite these limitations, this study has practical and theoretical implications. The concepts of power, authority, and influence have received scant empirical attention in implementation science. This research highlights the mechanisms by which these micropolitical contextual features influence successful implementation. Due to their influence, gaining support across multiple leadership levels is necessary to disseminate broadly and reinforce the importance of an intervention among staff. However, the hierarchical structure of MDTs will impact how team members perceive the intervention. Therefore, future studies must engage each discipline in discussions about implementation and tailor communication to assure all interest groups understand the value and utility of the intervention relative to their role. By advancing the understanding of these power dynamics, future researchers can develop appropriate implementation strategies to account for the micropolitical context, increasing staff engagement in change efforts.

The omission of micropolitics from implementation theories may explain why the contextual determinants of power, authority, and influence are largely absent from the extant literature. Theories within implementation science are invaluable for identifying contextual influences, and predicting how implementation may progress [87]. Although the use of theory in implementation science has increased over time, engagement with theoretical knowledge remains a one way process [38]. Theories mostly inform data collection and analysis, aiding researchers to identify similarities between their empirical findings and an extant theory [38]. However, deductive analysis using determinants within an established theory risks prematurely excluding alternative ways of organising the data that may reveal more novel findings [88]. This research highlights the value of using theory as a tool to be improved rather than a prescriptive checklist. This study’s inductive approach facilitated the identification of additional contextual variables associated with the micropolitics of healthcare not explicitly acknowledged within the CFIR [23]. Accounting for these concepts within the developed conceptual framework (Fig. 1) will support researchers when implementing change in routine practice.

## Conclusion

This study introduces the micropolitical concepts of power, authority, and influence as essential contextual determinants and outlines the mechanisms through which these concepts impact implementation within healthcare teams. Although the importance of these concepts has been previously recognised in organisational theory, micropolitics has received scant attention in implementation science. By providing a novel insight into the dynamic impact of power, authority, and influence, change agents can develop more appropriate implementation strategies to create contexts receptive for change. Furthermore, this research has developed a framework to capture the multidimensional influence of politics and power on implementation success. This valuable knowledge will help researchers negotiate the everyday politics of healthcare to support the successful implementation of evidence in routine practice.

## Supplementary Information

**Additional file 1: Supplementary file 1.** Description of the implemented collective leadership intervention and researcher reflexivity.

**Additional file 2: Supplementary file 2.** Observation template.

**Additional file 3: Supplementary file 3.** Interview guide.

**Additional file 4: Supplementary file 4.** Context coding framework- Adapted from Rogers et al. [54] (http://creativecommons.org/licenses/by/4.0/).

## Data Availability

Data sharing is not possible to protect the confidentiality of participants and to align with the ethical approval received from the University College Dublin Research Ethics Committee (ref: HREC-LS-16-116397).

## Abbreviations

AHP: Allied Healthcare Professional
CNM: Clinical nurse manager
CL: Collective leadership
CFIR: Consolidated Framework for Implementation Research
HCP: Healthcare professional
MDT: Multi-disciplinary team
