# Supplementary material for: The micropolitics of implementation; a qualitative study exploring the impact of power, authority, and influence when implementing change in healthcare teams
Source: BMC Health Serv Res. 2020 Nov 23;20:1059. doi: 10.1186/s12913-020-05905-z (PMC7684932; doi:10.1186/s12913-020-05905-z)
Supplement: Supplementary file 1 — Additional file 1: Supplementary file 1. Description of the implemented collective leadership intervention and researcher reflexivity. [file 12913_2020_5905_MOESM1_ESM.docx]

The healthcare initiative implemented is an educational intervention. This intervention aims to introduce collective leadership to healthcare teams to improve team performance and safety culture (1). Rather than targeting the leadership competencies of a single heroic leader using an existing training framework, the intervention aims to develop all team members’ leadership capabilities using an intervention driven by the needs of healthcare professionals. To achieve this, the team utilised a co-design approach to develop and prioritise content for the intervention (2). The co-design process involved health system researchers working collaboratively with individuals employed within the health system (healthcare professionals), and individuals who have experience using the system (patient representatives) over six-monthly three-hour workshops.

Through using the co-design approach, a toolkit of collective leadership interventions (a series of one-hour team sessions) was created (3). This toolkit is grounded in the realities of modern healthcare as it was developed based on the expertise, experiences, and reported needs of the co-design team members (2). The developed interventions include six foundational components that are compulsory for each team to complete and an additional 13 targeted interventions which teams can select based on their perceived needs and team priorities. This flexibility is reflected by the teams participating in this research. Although many of the targeted intervention sessions were comparable across cases (e.g. implementing a structured communication tool), some team choices varied to reflect the diverse goals of each team (e.g. improving psychological safety vs collaboratively deciding key performance indicators). While each intervention component is informed by the extant literature, three of these team sessions are structured around established healthcare tools. The remaining interventions are designed specifically to respond to the needs of MDTs as identified during the co-design process. Each intervention is one-hour long and is implemented on (at least) a monthly basis by teams in their own working environment. All intervention materials are open access via the project website and include topics such as goal setting, role clarity and removing frustration within the team (3).

LR completed each phase of data collection for this research. This researcher is a doctoral student who has received training in qualitative research methods from her supervisors (ADB and EMA) and through the completion of university modules. As a registered nurse, LR has prior experience working within multidisciplinary healthcare teams. However, this researcher was unfamiliar with the two cases (teams) of study, which heightens her ability to make the familiar strange, increasing analytical distance from the setting. As qualitative data collection and analysis relies heavily on the researcher’s interpretation of events (4), LR’s identity has influenced this research. However, how or, to what is difficult to predict. However, the reflexive journal maintained by LR helped to map this influence and ensure transparency in the data collected and during the analytic process. Additionally, consultation with the Co-Lead research team and regular feedback are additional strategies employed to mitigate this impact.

**References**

1. McAuliffe E, De Brún A, Ward M, O’Shea M, Cunningham U, O’Donovan R, et al. Collective leadership and safety cultures (Co-Lead): Protocol for a mixed-methods pilot evaluation of the impact of a co-designed collective leadership intervention on team performance and safety culture in a hospital group in Ireland. BMJ Open. 2017;7.

2. Ward M, De Brún A, Beirne D, Conway C, Cunningham U, English A, et al. Using Co-Design to Develop a Collective Leadership Intervention for Healthcare Teams to Improve Safety Culture. International Journal of Environmental Research and Public Health. 2018;15:1–17.

3. Co-Lead. Collective Leadership and Safety Cultures Toolkit [Internet]. Collective Leadership and Safety Cultures. 2019. Available from: https://www.ucd.ie/collectiveleadership/resourcehub/toolkit/

4. Darke P, Shanks G, Broadbent M. Successfully completing case study research: combining rigour, relevance and pragmatism. Info Systems J. 1998;8:273–89.
