# Supplementary material for: The micropolitics of implementation; a qualitative study exploring the impact of power, authority, and influence when implementing change in healthcare teams
Source: BMC Health Serv Res. 2020 Nov 23;20:1059. doi: 10.1186/s12913-020-05905-z (PMC7684932; doi:10.1186/s12913-020-05905-z)
Supplement: Supplementary file 2 — Additional file 2: Supplementary file 2. Observation template. [file 12913_2020_5905_MOESM2_ESM.docx]

| **Observation template** |
| --- |
| **Team:** |
| **Date: Time: Location:** |
| **Description of first impressions:**   - *Physical Environment (size, shape, noise, equipment)* - *Reach (number, disciplines, first time attendees vs returning)* - *General Context (e.g. busyness of the ward, priorities to other projects)* |
| **Description of the Intervention:**  *1) What type of atmosphere is there, formal/informal (does this change as the intervention progresses)?*  *2) How was the project/intervention introduced (by facilitator vs researcher)?*  *3) Did the introduction include a summary of what has been achieved so far?*  *4) Who was the facilitator, how did they deliver the intervention (nervous, confident)?*  *5) What were the key intervention events (steps)? Was this delivered as intended?*  *6) Were there any alterations made to the intervention (planned during preparation meeting vs unplanned)? If so, what were they?*  *7) Were there any conflicts? How were they resolved (by team vs researcher support)?*  *8) What were the team outputs?*  *9) Were participants actively engaged (who contributed/didn’t contribute)?*  *10) Did it appear as if participants enjoyed the intervention?*  *11) Did any aspect of the implementation process cause confusion (requiring researcher support)?*  *12) Were there any incentives to attend (e.g. hot food, CPD points (how many people registered for same))?*  *13) Is it evident that the intervention has been prepared (handouts printed etc.)?*  *14) Was the intervention evaluated? If so, how many took part?*  *15) Was there a debriefing session or follow up session following the intervention to discuss the outcome of the intervention?* |

| **Implementation Outcomes Proctor et al. (37)** | **Definition** | **Question Number** |
| --- | --- | --- |
| Acceptability | Satisfaction with the intervention (I like the intervention, it is appealing, I approve, I welcome it) | 1, 9, 10 |
| Fidelity | Adherence, delivered as intended | 2,4,5,6,9 |
| Feasibility | Suitability of the intervention for everyday use (the intervention is implementable, doable, easy to do/use) | 8, 13 |
| Adoption | Uptake, utilisation | 9, 13 |
| Appropriateness | Compatibility, relevance (the intervention’s suitable, applicable, a good fit) | 4, 8, 9, 11 |
| Cost | Cost of the intervention, the implementation strategy used and location of delivery | 12 |
| Penetration | Reach, level of institutionalisation | Preliminary Question (Reach) |
| Sustainability | Maintenance, routinisation |  |
| **CFIR Domain -Damschroder et al. (39)** | | **Question number** |
| **Intervention Characteristics**- *internally vs externally developed, adaptability, complexity, cost, strength of evidence* | | 6 (adaptability) |
| **Outer Setting**- *patient’s needs, degree to which the organisation is networked to other organisations, competitive pressure to implement (competing organisation), external incentives (guidelines)* | | Preliminary Q (general context) |
| **Inner Setting**- *age, maturity, size of the organisation, team stability, communication, culture, readiness for change* | | 1, 7, 9, 10 |
| **Individual Characteristics**- *knowledge and belief about the intervention, belief in own capabilities, personal traits (motivation, values, intellectual ability, tolerance, ambiguity)* | | 4, 10, 12 |
| **Implementation Process**- *planning and executing the intervention, engaging, and involving the appropriate individuals, reflection, and evaluation (debriefing)* | | 2, 3, 5, 6, 8, 9, 11, 13, 14, 15 |
