# Supplementary material for: The micropolitics of implementation; a qualitative study exploring the impact of power, authority, and influence when implementing change in healthcare teams
Source: BMC Health Serv Res. 2020 Nov 23;20:1059. doi: 10.1186/s12913-020-05905-z (PMC7684932; doi:10.1186/s12913-020-05905-z)
Supplement: Supplementary file 3 — Additional file 3: Supplementary file 3. Interview guide. [file 12913_2020_5905_MOESM3_ESM.docx]

**Implementation Evaluation**

**Aim**: to understand how the intervention has been implemented

*Introduce self, explain research aims briefly, check interviewee has read the information sheet, answer any questions, ask interviewee to sign consent form and advise they can keep copy of information and/or consent sheets. Request permission to record - explain processes.*

**Background questions**

- Can you tell me a bit about your professional background / time as a X professional?
- Time in current role / time in current team
- Can you briefly tell me about your current role and responsibilities in this team (NB the intervention team)?

**Collective leadership intervention implementation**

1. How did you hear about the collective leadership intervention?
2. Thinking back to before the collective leadership intervention began can you remember what you (and/or your team) expected from the team sessions?
3. Were you involved in the team sessions in any capacity (as an organiser/facilitator or attendee)? *Probe: There were 8 sessions; can you remember how many you attended?*
4. What influenced your decision or ability to attend (or not attend) the team session(s)?
5. Can you tell me about the typical attendance levels at the sessions?

- *Did the attendance levels change over time? Why?*
- *Do you think there was appropriate attendance from all disciplines? Why/why not?*

1. What was your impression of the sessions? *What worked well and what didn’t work so well? Were the sessions relevant to you? Were the sessions enjoyable to attend?*
2. Do you think that the collective leadership intervention worked for your team? *What makes you say this? Why was this the case do you think? How did the sessions lead to that outcome?*
3. Do you think there has been any change in how the team are working or working together as a result of the collective leadership intervention? *What makes you say that? What has changed or what has been initiated through the team’s involvement in the collective leadership intervention? What impact have these changes had (on staff, patients, team performance)?*
4. If you’re currently working with other teams, have you spread/shared anything you learned through the collective leadership intervention with this team and applied it to your work with other teams or colleagues?
5. Do you think any components of the team sessions that you implemented will be sustained and continued by the team? *Why do you say this? What will the challenges to sustaining this in the team?*

| **Interview Question** | **Implementation outcome** | **CFIR domain** | |
| --- | --- | --- | --- |
|  |  | *Inner setting* | - Networks and communication - Leadership engagement - Access to information and knowledge |
|  | - Appropriateness | *Intervention characteristics* | - Relative advantage |
|  |  | *Inner setting* | - Relative priority |
|  |  |  | |
|  | - Acceptability - Feasibility | *Inner setting* | - Culture - Leadership engagement - Networks and communication - Organisational incentives |
|  |  | *Implementation process* | - Champions |
|  | - Penetration |  | |
|  | - Acceptability - Appropriateness - Feasibility | *Intervention characteristics* | - Relative advantage |
|  | - Acceptability - Appropriateness - Adoption - Feasibility - Penetration | *Intervention characteristics* | - Relative advantage |
|  |  | *Inner setting* | - Structural characteristics - Available resources |
|  | - Penetration | *Intervention* *characteristics* | - Relative advantage |
|  |  | *Inner setting* | - Structural characteristics |
|  | - Penetration | *Inner setting* | - Networks and communication |
|  | - Sustainability |  | |
