# Supplementary material for: The micropolitics of implementation; a qualitative study exploring the impact of power, authority, and influence when implementing change in healthcare teams
Source: BMC Health Serv Res. 2020 Nov 23;20:1059. doi: 10.1186/s12913-020-05905-z (PMC7684932; doi:10.1186/s12913-020-05905-z)
Supplement: Supplementary file 4 — Additional file 4: Supplementary file 4. Context coding framework- Adapted from Rogers et al. [54] (http://creativecommons.org/licenses/by/4.0/). [file 12913_2020_5905_MOESM4_ESM.docx]

Context coding framework-Adapted from Rogers et al.{49} ([**http://creativecommons.org/licenses/by/4.0/**](http://creativecommons.org/licenses/by/4.0/)**)**

| **System Level** | **Characteristic** | **Definition** | | **Example of Supporting Data** | **Construct Rating Examples**  **(positive, neutral, negative influence on implementation)** |
| --- | --- | --- | --- | --- | --- |
| System-Level Determinants | Social Environment | Cosmopolitanism | How connected the hospital is with external organisations/events and the impact of this network. | View that being affiliated with a hospital group (hospitals in Ireland organised into seven hospital groups) provides more learning opportunities for staff. | +2 |
|  |  | Peer Pressure | Mimetic or competitive pressure to implement an intervention. | A team member asks researchers if other teams have “embedded it better” and how this was achieved. | +1 |
|  | Political Environment | External Incentives and Influence | External incentives to spread the uptake of interventions (national policy, guidelines, collaborations), external influence regarding decision making (e.g. external change agents). | Perceived threat following a proposed systems change leaving staff anxious about future prospects. | -1 |
|  | Economic Environment | External economic factors within the wider health system which may influence the capacity and resources available to the setting. | | Disparity in funding. Hospitals, comparable in size and characteristics acknowledged as receiving greater resources due to previously publicised incidents. | -2 |
| Organisational-Level Determinants | Structural characteristics | Hospital Classification | | Participants confirm an increased demand on the hospital, with the number of patients on trolleys exceeding the norm. | -2 |
|  |  | Hospital size | |  |  |
|  |  | Hospital workload | |  |  |
|  | Networks and communications | The quality of communication within the organisation and relationships amongst its members. | | National survey data highlight strong relationships among staff, however, relationships between management and frontline appear taut. | 0 |
|  | Culture | The norms, values and assumptions of the organisation, the degree of autonomy given to staff and their perceptions of change. | | Hospital documents suggest a culture characterised by openness, trust and inclusion. | +2 |
|  | Compatibility | Is there a tangible fit between the values and norms of the organisation to the intervention? | | The collective leadership intervention appears to align with the open culture outlined in hospital reports. | +2 |
|  | Organisational support | Is organisational support evident? Are rewards offered by the organisation for engagement with the intervention? | | Food provided by the organisation at each session and is suggested to enhance staff attendance. | +1 |
|  | Organisational climate | Staff perceptions of and emotional responses to the characteristics of their organisation including attitudes towards learning. | | One team member discusses the importance of valuing staff by supporting their educational needs. | +2 |
|  | Organisational leadership engagement | Are organisational leaders/managers (e.g. CEOs, executive members) committed/to the implementation effort? | | Senior managers:   - Encouraged engagement - Ensured follow through with outcomes - Provided resources - Organised implementation | +2 |
|  | Available resources | The level of resources available within the organisation to complete the intervention including human (e.g. appropriate staffing levels), financial and technological resources. | | Noted that if one team member “was left do his job, the hospital would benefit but it doesn’t have the resources”. | -1 |
| Team-Level Determinants | Structural characteristics | Team size | | Workload: participant notes she had “no time” to prepare for the intervention, it “makes up 0.001% of our work”. | -2 |
|  |  | Team turnover/stability | |  |  |
|  |  | Team workload | |  |  |
|  | Teamwork | The quality of communication within the team and relationships amongst its members. | | “Unless you approach {them} you would get no communication throughout the day”. | -2 |
|  | Culture | The norms, values and assumptions of the team, the degree of autonomy given to staff and their perceptions of change. | | “Put up and shut up” mind set  “…views are valued, sought out in comparison to other multidisciplinary teams I would have been on…like every member is valued and their input is welcomed”. | 0 |
|  | Compatibility | Does the intervention fit with existing workflows of the team? | | Due to the “pressurised” nature of the ward environment (high patient turnover and poor staffing levels) the compatibility of intervention with the team’s current workload is questionable. | -2 |
|  | Available resources | The level of resources available to complete the intervention within the team including human (e.g. appropriate staffing levels), financial and technological resources. | | Inadequate staffing impeded staff engagement with the intervention: “we were short staffed, just couldn’t get the time”. | -2 |
|  | Local leadership engagement | Are frontline leaders/managers (e.g. consultants, clinical nurse managers) committed and involved in the implementation? Are peer leaders evident? | | One senior team member asks to take intervention materials to use with junior doctors at another education session. | +1 |
|  | Team efficacy | Does the team believe in their skills and capabilities to implement the intervention successfully? | | The team raise concerns regarding lack of training and skills to achieve their developed goals. | -1 |
| Individual-Level Determinants | Self -efficacy | An individual’s belief in their capabilities to implement the intervention and manage its outputs. | | One team member indicates that he is capable to contribute more to the team, but his job role does not allow this. | -1 |
|  | Individual attitudes | Participants perceptions of the advantage and relevance of the intervention. Is the intervention’s implementation considered a priority or an additional burden in daily practice? | | The intervention is “a great way of stopping and reflecting”. | +2 |
